# Retrospective assessment of tolerability and efficacy of zoledronate in the palliative treatment of cancer‐bearing dogs

**DOI:** 10.1111/avj.13218

**Published:** 2022-11-16

**Authors:** MG Lopes, G Tosi, KA McNaught, JS Morris

**Affiliations:** ^1^ School of Veterinary Medicine, Small Animal Hospital, College of Medical, Veterinary, and Life Sciences University of Glasgow Glasgow UK

**Keywords:** bisphosphonates, canine, hypercalcaemia, neoplasia, tumour

## Abstract

Zoledronate is a bisphosphonate frequently used for the treatment of hypercalcaemia of malignancy and tumour‐associated bone pain in dogs, however, there is a paucity of information regarding its use in veterinary medicine. The aim of this retrospective study was to report the tolerability of zoledronate in the palliative treatment of cancer‐bearing dogs and secondarily to to assess the efficacy of zoledronate for the treatment of hypercalcaemia of malignancy. Thirty‐seven dogs (22 with tumour‐associated bone pain and 15 with hypercalcaemia of malignancy) that received 114 zoledronate infusions were included. Tolerability was assessed by the absence of post‐zoledronate hypocalcaemia or other adverse events as defined by Veterinary Cooperative Oncology Group‐Common Terminology Criteria for Adverse Events criteria. Efficacy was assessed by comparison of available ionized calcium levels before and after zoledronate administration in hypercalcaemic dogs. In 79% of zoledronate infusions, no adverse events were reported. The majority of adverse events which occurred in the other 21% of infusions could be attributed to concurrent chemotherapy or the underlying neoplastic disease. There was a small but significant increase in creatinine following treatment with zoledronate, however, none of the dogs developed clinically significant renal disease. In eight hypercalcaemic dogs with available ionized calcium following zoledronate administration, ionized calcium decreased rapidly within 7 days following treatment with zoledronate. Zoledronate is well‐tolerated with few recorded adverse events, however, monitoring of serum creatinine is advised. Zoledronate seems to be effective in the treatment of hypercalcaemia of malignancy.

**Abbreviation**
VCOG‐CTCAE: Veterinary Cooperative Oncology Group‐Common Terminology Criteria for Adverse Events

Bisphosphonates are a group of synthetic pyrophosphate analogues that exert their pharmacologic effect by inhibiting osteoclastic activity.^1^ This makes them useful in a range of metabolic and metastatic bone diseases and in human medicine, they are used in the treatment of osteoporosis, Paget's disease of bone, palliation of bone metastasis and paraneoplastic hypercalcaemia.^2^, ^3^ In veterinary medicine, bisphosphonates are used similarly for the treatment of idiopathic hypercalcaemia, hypercalcaemia of malignancy and tumour‐associated bone pain of primary or metastatic bone tumours.^1^ Anti‐neoplastic properties have also been suggested in humans, experimental animals^4^, ^5^, ^6^, ^7^, ^8^ and in the veterinary literature.^9^, ^10^

Pamidronate is widely used in the treatment of canine and feline hypercalcaemia of malignancy and tumour‐associated bone pain,^11^, ^12^, ^13^, ^14^ however, zoledronate has become increasingly popular in recent years. Zoledronate is a potent, third‐generation amino‐bisphosphonate, with an *in vitro* antiresorptive potency 100 times that of pamidronate.^15^ It is also less nephrotoxic than pamidronate in humans^16^ and mice,^17^ and can be administrated over a shorter duration (15 min vs 2 h), which makes it more convenient in the clinical setting. It is generally well‐tolerated, but some adverse events have been identified in humans, such as osteonecrosis of the jaw,^18^ and renal toxicity. Jaw osteonecrosis was also reported in a single case of a dog with appendicular osteosarcoma treated with zoledronate for a prolonged period of time.^19^ Renal toxicity, although rare, has been reported in humans,^20^ mice^21^ and in a recent study of dogs treated with zoledronate for malignant osteolysis.^22^ In this study, 15.9% of dogs (n = 7) developed acute kidney injury (AKI), however, it was only felt likely to be secondary to the zoledronate in two dogs (4.6%).^22^

Although some studies report the use of zoledronate in dogs with naturally occurring bone tumours,^19^, ^22^, ^23^ there is still a paucity of literature reporting the use of zoledronate in cancer‐bearing dogs. A recent study by Brewer et al^24^ evaluating the toxicity of zoledronate in the treatment of dogs with bone pain or hypercalcaemia concluded zoledronate is well tolerated, even when multiple doses were administered over time. This study also evaluated the efficacy of zoledronate in the treatment of 9 hypercalcaemic dogs, and together with a small case series of 4 dogs,^25^ represent the only reports of zoledronate for the treatment of canine hypercalcaemia. The aim of this retrospective study was to enhance the literature by reporting the tolerability of zoledronate in the treatment of 37 cancer‐bearing dogs. A secondary goal was to report the efficacy of zoledronate in the treatment of hypercalcaemia of malignancy.

## Materials and methods

For this retrospective study, medical records of dogs that presented to University of Glasgow Small Animal Hospital between November 2014 and May 2021 were reviewed to retrieve those that had received zoledronate. Dogs that had a cytological, histopathological or a strong presumptive imaging diagnosis of neoplasia (based on imaging studies reported by a board‐certified diagnostic imager or by a resident working under the supervision of a board‐certified diagnostic imager) treated with at least one dose of zoledronate for hypercalcaemia of malignancy or tumour‐associated bone pain were included. Dogs were diagnosed with hypercalcaemia of malignancy if they were found to have an ionized calcium >1.50 mmol/L (reference interval 1.25–1.50) concurrently with a diagnosis of neoplasia at any point during the investigation or treatment of their disease. For inclusion in the study, dogs were also required to have a minimum baseline database which included complete clinical history, physical examination, haematology, biochemistry and staging with thoracic and abdominal imaging. Information on additional treatments such as surgery, radiotherapy, chemotherapy and analgesics was also retrieved from the records. Dogs were excluded if they had received zoledronate for the treatment of a non‐neoplastic disease or if there was no follow‐up after zoledronate infusion to allow assessment of adverse events or post‐zoledronate ionized calcium in dogs with hypercalcaemia of malignancy.

Assessment of tolerability was in three ways: (1) Adverse events reported by the owners following treatment with zoledronate were retrieved from the records, recorded, and graded according to the Veterinary Cooperative Oncology Group‐Common Terminology Criteria for Adverse Events (VCOG‐CTCAE v2)^26^; (2) plasma creatinine before and after treatment was assessed as a measure of renal function and nephrotoxicity, using International Renal Interest Society (IRIS) guidelines for reference ranges: normal creatinine was defined as creatinine <125 umol/L, mild azotaemia 125–250 umol/L, moderate azotaemia 251–440 umol/L and marked azotaemia >440 umol/L. AKI was graded according to the IRIS guidelines for grading for AKI: grade I AKI was defined as an increase in creatinine >26.4 umol/L within the normal reference range (creatinine <140 umol/L), grade II AKI was defined as an increase in creatinine >26.4 umol/L to a final value of creatinine between 141 and 220 umol/L and grade III AKI was defined as an increase in creatinine >26.4 umol/L to a final value of creatinine between 221 and 439 umol/L; and (3) baseline ionised calcium was examined to assess if dogs became hypocalcaemic following zoledronate administration.

Assessment of efficacy in the treatment of hypercalcaemia of malignancy was performed by comparing baseline ionized calcium levels with ionized calcium levels within 1 week of treatment with zoledronate, when available.

Plasma biochemistry and ionized calcium were routinely collected before each treatment with zoledronate for assessment of nephrotoxicity and hypocalcaemia. Biochemistry was run on Catalyst Dx (IDEXX Laboratories Pty Ltd, 2010). Ionised calcium was run in house on VetStat Electrolyte and Blood Gas Analyzer (IDEXX, 2010). Normal reference range for ionised calcium was 1.25–1.50 mmol/L.

Zoledronate infusions were administered as a continuous rate intravenous infusion diluted with saline (25 mL for dogs under 10 kg and 100 mL for dogs over 10 kg) over 15 min. Prescribed dose range was between 0.1 and 0.25 mg/kg with a maximum dose of 4 mg per infusion. Repeat infusions were scheduled every 3–4 weeks.

## Statistical analysis

The statistical analysis was performed using commercial software (Minitab 19), with statistical significance set at P ≤ 0.05. Paired variables were analysed using parametric (paired t‐test) and non‐parametric (two‐sample Wilcoxon signed‐rank test) testing, depending on the normality of data distribution, which was assessed using a Ryan‐Joiner normality test. Data are reported as mean ± standard deviation for normally distributed data and median with range for non‐parametric data.

For analysis of creatinine values, ‘baseline creatinine’ was defined as creatinine values before any zoledronate had been administered, and ‘post‐treatment creatinine’ as the highest creatinine retrieved from the records after the first zoledronate dose.

For analysis of ionized calcium values, ‘pre‐treatment ionized calcium’ was defined as ionized calcium taken before every zoledronate infusion.

## Results

The records of 44 dogs that received zoledronate were retrieved. One dog was excluded as it had received zoledronate for hypercalcaemia secondary to granulomatous disease, one dog was excluded as it had received zoledronate for hypercalcaemia secondary to vitamin D toxicity, and five dogs were excluded as they were euthanised after a median of 3 days post‐zoledronate infusion (range 2–7 days) with no follow‐up data regarding possible adverse events. Thirty‐seven dogs met the inclusion criteria. Mean age at diagnosis was 9 years (range 4–14 years). Twenty‐two dogs were male (14 neutered) and 15 dogs were female (11 neutered). The mean weight was 30.3 kg (range 8.0–64.2 kg). The most common breed was Labrador retriever (n = 9), followed by Collies (n = 5), Golden retriever (n = 5), Cocker spaniel (n = 2), Crossbreed (n = 2), and one each of the following breeds: Springer spaniel, Greyhound, Rodhesian Ridgeback, Bernese Mountain Dog, Boxer, Dogue de Bordeux, Rottweiller, Staffordshire bull terrier, Leonberger, Cavalier King Charles Spaniel, Labradoodle, West Highland White terrier, Bichon Frise and German Shepherd Dog.

Tumours types included osteosarcoma (n = 9), anal sac adenocarcinoma (n = 9), soft tissue sarcoma with bone invasion (n = 3), lymphoma (n = 3), vertebral tumour (n = 2), multilobular osteochondrosarcoma (n = 2), thyroid carcinoma (n = 1), metastatic carcinoma with unknown primary tumour (n = 1), digital squamous cell carcinoma (n = 1), frontal sinus carcinoma (n = 1), metastatic pulmonary carcinoma (n = 1), multiple myeloma (n = 1), insulinoma (n = 1), pelvic neuroendocrine tumour (n = 1) and a metastatic humeral proliferative bone lesion (n = 1).

Ten dogs (27%) underwent surgery, either definitive intent or palliative, at some point during the course of their treatment; seven dogs (19%) received radiotherapy either before, concurrently or after zoledronate treatment; and 13 (35%) received chemotherapy or tyrosine kinase inhibitors (TKI) either before, concurrently or after zoledronate infusions. Six dogs (16%) were treated with a combination of surgery and chemotherapy or TKI; two dogs (5%) were treated with surgery and radiotherapy; one dog (3%) was treated with surgery, radiotherapy, and chemotherapy. Other concurrent medications included non‐steroidal anti‐inflammatory drugs (NSAIDs) in 27 dogs (73%), corticosteroids in six dogs (16%) and other pain relief medication in 26 dogs (70%) (paracetamol, gabapentin, tramadol, buprenorphine, methadone).

A total of 114 zoledronate infusions were administered during the study period, with a median of two infusions per dog (range 1–8), a median dose per infusion of 0.1 mg/kg (range 0.062–0.25 mg/kg) and median interval between infusions of 4 weeks. Sixty‐three zoledronate infusions were administered to the 22 dogs with tumour‐associated bone pain and 51 infusions were administered to the 15 dogs with hypercalcaemia of malignancy.

Three dogs were still alive at the time of data analysis. Median survival time was 122 days (range 5–462 days). The cause of death/euthanasia for all dogs was related to their neoplastic disease.

### Tolerability of zoledronate

#### Adverse events

After 90/114 (79%) zoledronate infusions no adverse events were reported. Adverse events reported in the remaining 24 zoledronate infusions, which affected 15 dogs, are summarised in Table 1. The most commonly reported adverse events included diarrhoea, AKI, lethargy and oral bleeding. A total of 32 adverse events were noted with the majority being grade 1 and 2 according to the VCOG‐CTCAE. For 17/32 (53%) of adverse events reported following zoledronate, the dog was also receiving some form of chemotherapy, but 50% (16/32) of adverse events reported could also be attributed to the dog's neoplastic disease or concurrent comorbidities, and for 6 of these, the dogs were also receiving chemotherapy.

**Table 1:** Veterinary Cooperative Oncology Group‐Common Terminology Criteria for Adverse Events recorded following treatment with zoledronate per infusion

Adverse events		Grade 1	Grade 2	Grade 3	Grade 4	Grade 5
Gastrointestinal						
Diarrhoea		7	1			
Constipation		2				
Altered appetite		2				
Constitutional						
Lethargy		2	1			
Musculoskeletal						
Lameness		2				
Haemorrhage						
Oral bleeding		3				
Neurology						
Ataxia				2		
Tremors		1				
Renal						
Acute kidney injury		2	2			
Proteinuria				2		
Metabolic						
Hypocalcaemia					1	
Dermatological						
Oedema (muzzle)		1				
Cardiac general						
Cardiopulmonary arrest						1

One dog suffered a cardiac arrest and died the day after the second zoledronate infusion. This dog was being treated with toceranib for metastatic anal sac adenocarcinoma and developed hypercalcaemia of malignancy (ionized calcium of 1.82 mmol/L), and neck pain with disease progression. A presumptive diagnosis of paraneoplastic neuritis was made following advanced imaging (full body CT and cervical MRI). After the first dose of zoledronate, clinical signs associated with hypercalcaemia of malignancy improved, however, 3 weeks later prior to the second dose, the hypercalcaemia of malignancy had recurred (ionized calcium of 2.63 mmol/L). Cardiac arrest occurred 24 h after the second dose of zoledronate infusion. Both electrolytes and ionized calcium were checked during cardiopulmonary resuscitation and while electrolytes were normal, the ionized calcium remained elevated (2.07 mmol/L).

#### Creatinine levels

Plasma creatinine concentration was used as an estimate of nephrotoxicity following zoledronate administration. Creatinine values were available for 29 dogs both before and after treatment. At baseline, 24 dogs had normal creatinine levels while four dogs had mild azotaemia and one dog had moderate azotaemia. The median pre‐treatment creatinine was 88 umol/L (range 44–284 umol/L). There was a small but significant difference between baseline creatinine and post‐treatment creatinine in the 29 dogs (P = 0.043), with the median highest creatinine post‐treatment being 94 umol/L (range 43–252 umol/L). However, there was no significant increase in creatinine levels noted in the 5/29 dogs with baseline creatinine above the reference range (median baseline 169 umol/L vs post‐treatment 178 umol/L, P = 0.89).

Since concurrent radiotherapy and repeated anaesthesia could affect renal function, the creatinine in these dogs was examined separately. Post‐zoledronate creatinine was available for 6/7 dogs that received radiotherapy (median of six fractions, range 4–12 fractions per patient). Five dogs had normal creatinine and one dog had mild azotaemia prior to treatment with zoledronate. Creatinine was significantly increased post‐treatment (median 90 umol/L) compared to baseline (median 72 umol/L) in this group (P = 0.031).

Twenty out of twenty‐nine dogs received NSAIDs as a part of their treatment. Since this could also have affected renal function, the creatinine in these dogs was examined separately. There was also a significant difference between baseline (median 83.5 umol/L) and post‐treatment creatinine (91.5 umol/L) in this group (P = 0.027).

Creatinine was also evaluated separately in 13/29 dogs, which were hypercalcaemic on presentation as this may also have affected renal function. There was no significant difference between baseline (97 umol/L) and post‐treatment creatinine (112 umol/L) in this group (P = 0.88).

Overall, four dogs (13.7%), which were all non‐azotaemic prior to zoledronate, experienced increases in creatinine following zoledronate consistent with AKI: two dogs experienced grade I AKI, and two dogs experienced grade II AKI. Both dogs that had grade I AKI were also treated with radiotherapy and NSAIDs; in one of the dogs, the AKI was detected 5 months after the first dose of zoledronate and 3 months after completion of the radiotherapy protocol, in the other dog, the AKI was detected 2 months after the first of two doses of zoledronate and 1 month after completion of the radiotherapy protocol, but later returned to baseline values 2 months after the AKI was detected. The two dogs that experienced a grade II AKI received 8 and 4 doses of zoledronate, and the AKI was detected after dose 3 and 1, respectively. One of the dogs was receiving additional treatment which included toceranib and NSAIDs and its creatinine normalised; the other dog received no additional treatment and its creatinine remained stable, with no evidence of clinical azotaemia.

#### Calcium levels in dogs

Ionised calcium was examined in all dogs prior to treatment with zoledronate to assess for evidence of symptomatic hypocalcaemia, which was approximately 3–4 weeks after the previous zoledronate infusion for patients receiving more than one zoledronate infusion. Ionized calcium levels taken immediately before treatment were available for 57/63 zoledronate infusions given to dogs treated for tumour‐associated bone pain. The mean ionized calcium before zoledronate treatment was 1.24 mmol/L (±SD 0.05), with hypocalcaemia (ionized calcium <1.25 mmol/L) documented prior to 28/57 infusions. Twelve dose delays of one week were prescribed at the clinician's discretion due to ionized hypocalcaemia (mean 1.15 mmol/L ± 0.06), however, no patient was ever symptomatic for the hypocalcaemia. One dog with tumour‐associated bone pain was prescribed oral calcium supplementation for the duration of its zoledronate treatment at the clinician's discretion.

Ionized calcium levels taken immediately before treatment were available for all 51 zoledronate infusions given to hypercalcaemic dogs. The mean ionized calcium before treatment with zoledronate was 1.94 mmol/L ± SD 0.32. No patient was found to be hypocalcaemic prior to treatment with zoledronate, however, a single episode of clinical hypocalcaemia (grade 4) was noted, and this occurred in a dog with hypercalcaemia of malignancy secondary to relapsed high‐grade T‐cell lymphoma. The dog presented in poor clinical condition with an ionized calcium of 2.39 mmol/L which failed to respond to intravenous fluid therapy and dexamethasone, so in an effort to reduce the ionized calcium levels, both L‐asparaginase and zoledronate were given 48 h apart. Although 2 days following zoledronate the ionized calcium was 1.02 mmol/L and the dog remained asymptomatic, by day 5 the dog represented with weakness and muscle fasciculations, and the ionized calcium had dropped further to 0.72 mmol/L. The dog was treated with intravenous and oral calcium and initially improved clinically, however, on day 7 the dog was euthanised due to clinical deterioration suspected secondary to the lymphoma. Four additional asymptomatic hypocalcaemic episodes were noted in dogs that had their ionized calcium levels checked in between zoledronate infusions, with values of 1.14, 1.16, 1.21 and 1.24 mmol/L recorded 4 days, 2 weeks, 2 days and 2 weeks after zoledronate infusions. The second dog received oral calcium supplementation at the clinician's discretion which led to recurrence of hypercalcaemia (1.63 mmol/L) 1 week later, but none of the other dogs received any supplementation.

### Efficacy of zoledronate in the treatment of dogs with hypercalcaemia of malignancy

**Figure 1:**
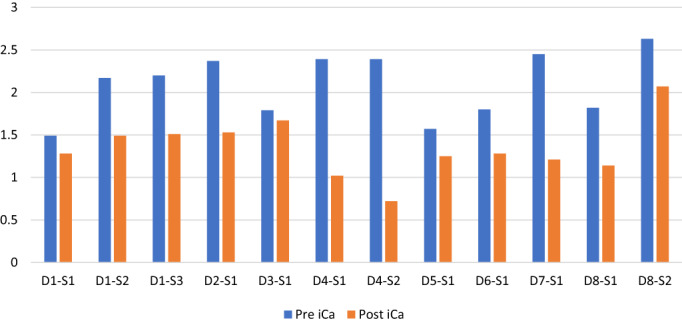
Plasma ionized calcium before zoledronate infusion and within the first 7 days from zoledronate infusion. D‐Dog; S‐Sample. Dog 1 had ionized calcium samples available pre and post‐zoledronate for three infusions, dog 4 and dog 8 had ionized calcium samples available pre and post‐zoledronate for two infusions, and dog 2, 3, 5, 6 and 7 had ionized calcium samples available pre and post‐zoledronate for one infusion. Pre iCa, ionized calcium prior to treatment with zoledronate; Post iCa, ionized calcium within 7 days post‐treatment with zoledronate.

Baseline ionized calcium values were available for all 51 zoledronate infusions given to treat the 15 dogs with hypercalcaemia of malignancy (Figure 1). The mean ionized calcium before treatment with zoledronate was 1.94 mmol/L ± SD 0.32. In 11/51 zoledronate infusions that were administered to a total of 8 hypercalcaemic patients, ionized calcium levels were available within the first seven days after the infusions. In one case the ionized calcium was checked twice during the first week after treatment, making a total of 12 ionized calcium results available within 7 days of zoledronate infusion. The ionized calcium for these samples had decreased by a median of 31% (range 7%–70%) after a median of 4 days (range 1–7 days). The median pre‐treatment ionized calcium for these 11 administrations was 2.17 mmol/L (range 1.49–2.63 mmol/L), and the median ionized calcium within the first week following treatment was 1.28 mmol/L (range 0.72–2.07 mmol/L) which was a significant decrease (P < 0.001) (Figure 1).

## Discussion

Zoledronate appears to be safe and well tolerated, with no adverse events reported following 79% of infusions, which is in line with what has been previously reported by Brewer et al (no adverse events in 89% of dogs).^24^ Although adverse events were recorded, they were mostly grade 1 or 2 in severity and, as the majority of patients were being treated with zoledronate alongside chemotherapy and/or analgesia (i.e. NSAIDs), it is possible the concurrent treatments have contributed to these adverse events. Additionally, most patients were being treated palliatively for advanced neoplastic disease, and some of the adverse events recorded could have also been caused by the patients' neoplastic disease. The most common adverse events in our study was diarrhoea. Gastrointestinal adverse events are commonly associated with chemotherapy and NSAIDs,^27^, ^28^ and although gastrointestinal irritation is a possible adverse event following oral administration of bisphosphonates in people,^29^ diarrhoea is not an adverse event commonly associated with zoledronate. In our cohort of dogs, all dogs that experienced diarrhoea during their treatment with zoledronate were being treated with chemotherapy, NSAIDs or had underlying gastrointestinal disease (such as inflammatory bowel disease), and as such these may have also contributed to the diarrhoea.

Renal toxicity and AKI, particularly in patients with pre‐existing renal disease have been reported in humans with zoledronate administration,^30^ and more recently also in dogs.^22^ In our study, there was a small but significant increase in creatinine levels following treatment compared to baseline, suggesting that zoledronate may impact renal function, however, most dogs in our cohort were also being treated with NSAIDs, which may have contributed to this difference. Four dogs (13.7%) experienced AKI, however, three dogs were receiving NSAIDs and two dogs underwent anaesthesia for treatment with radiotherapy. As such, the AKI may not be secondary to the zoledronate alone and it is possibly secondary to the combination of treatments. Additionally, the AKI was transient in two dogs, and as all dogs continued their treatment as planned, these results suggest that grade I and II AKI is unlikely to be of clinical significance in a cohort of dogs being treated in the palliative setting. Specific groups of dogs with additional risks factors could experience significant increases in creatinine levels, as seen in the small group of dogs undergoing multiple general anaesthetics for radiotherapy and the dogs receiving NSAIDs, however, without a control group it is not possible to know if these increases would have occurred regardless of treatment with zoledronate or if they occurred due to the combination of treatments. Prospective studies with a larger cohort of dogs and a control group would be required to clarify this. Interestingly, patients with pre‐treatment mild–moderate azotaemia or hypercalcaemic patients (who could be considered at higher risk of developing renal dysfunction) did not develop a significant increase in creatinine on follow‐up, which may be due to a type II error, but also suggests zoledronate should not be withheld from these patients if the benefits are expected to outweigh the risks. As the majority of our patients were being treated in the palliative setting for advanced/metastatic neoplastic disease, their survival times were short, so long‐term cumulative renal toxicity cannot be ruled out based on this study.

None of the dogs in our cohort experienced clinically apparent osteonecrosis of the jaw, although specific monitoring with CT of the head was not performed in any dog. Osteonecrosis of the jaw has been reported in a dog with osteosarcoma treated with monthly zoledronate for 46 months.^19^ Although two dogs experienced a total of three episodes of oral bleeding, which is a reported clinical sign of osteonecrosis of the jaw,^19^ both dogs had oral masses, which are far more likely to have caused the oral bleeding. One dog also experienced oedema of the muzzle, but this dog was also one of those with an oral mass, and the oedema resolved with antibiotics.

Hypocalcaemia following zoledronate treatment in hypercalcaemic patients is a possible adverse event in humans.^31^, ^32^, ^33^ Symptomatic hypocalcaemia only occurred in one hypercalcaemic dog that received definitive intent treatment for relapsed lymphoma. Thus, it may be prudent not to use zoledronate as a treatment for dogs with hypercalcaemia of malignancy for which definitive treatment of the primary tumour is planned in the near future.

In one patient, cardiac arrest occurred 24 h after treatment with zoledronate. Although we cannot rule out this was secondary to zoledronate administration, it is also possible it was secondary to the patient's advanced metastatic disease, persistent hypercalcaemia or suspected paraneoplastic neuritis.

Zoledronate also appears effective in the treatment of hypercalcaemia of malignancy. Ionized calcium levels reduced significantly within the first week following treatment (median 31% decrease) in the small number of cases where both pre and post‐treatment values were available. This is in line with what has been previously reported in a recent study by Brewer et al (median 40% decrease).^24^ Some of the hypercalcaemic patients were being treated with chemotherapy or tyrosine kinase inhibitors, which is a confounding factor for the efficacy of zoledronate, however, for most of these dogs, their neoplastic diseases were refractory to treatment, or the hypercalcaemia developed during the course of treatment. As such it is likely that most of the reduction in ionized calcium was due to the zoledronate rather than concurrent treatment, however, we cannot rule out that concurrent chemotherapy may have also played a role. Our results also imply a variable onset of action, as some dogs had a normal ionized calcium within 2–4 days of treatment, but three dogs remained mildly hypercalcaemic after 4–7 days. To the best of the authors knowledge, the duration of effect induced by zoledronate treatment in hypercalcaemia of malignancy also remains unclear, and prospective studies with serial measurements of ionized calcium levels following treatment with zoledronate would be required to establish this.

This is a retrospective study and has several limitations. Most patients were treated with either chemotherapy, radiotherapy, NSAIDs or a combination of these treatments alongside zoledronate, making it challenging to assess which of the observed adverse events can be reliably attributed to zoledronate. In our patient population, not all dogs had post‐treatment creatinine available and not all hypercalcaemic dogs had ionized calcium values measured within the first 7 days post‐treatment, which decreased the power of the analysis. We also had five dogs that were excluded from analysis due to euthanasia shortly after treatment with zoledronate and lack of follow‐up data and although this was felt most likely to be secondary to refractory pain and/or poor clinical condition secondary to the underlying neoplastic disease (metastatic histiocytic sarcoma, appendicular osteosarcoma, rib osteosarcoma, vertebral epithelial tumour and soft tissue sarcoma with rib invasion), we cannot completely exclude this was secondary to acute deterioration secondary to severe adverse events from zoledronate, such as AKI.

In conclusion, zoledronate is safe and well tolerated, and seems to be effective in the treatment of hypercalcaemia of malignancy. Symptomatic hypocalcaemia is a rare adverse event but can occur in dogs with hypercalcaemia of malignancy treated with zoledronate and that have also received definitive intent treatment for their underlying neoplastic disease. Clinically significant nephrotoxicity was not identified in this patient cohort, and although 13.7% dogs experienced AKI, this was transient in 6.9% dogs. Monitoring of biochemistry is advised particularly in patients undergoing concurrent treatments that may impact on renal function and are expected to experience prolonged survival times.

## Conflict of Interest and Sources of Funding

The authors declare no conflicts of interest or sources of funding for the work presented here.
